# Supplementary material for: Neuronal reactivation during post-learning sleep consolidates long-term memory in Drosophila
Source: eLife. 2019 Feb 25;8:e42786. doi: 10.7554/eLife.42786 (PMC6428568; doi:10.7554/eLife.42786)
Supplement: Supplementary file 1. — Table S1. LTM does not depend on the circadian time of training Suppression indices (SIs) of naïve (train-) and experienced (train+) males of the indicated genotypes according to Figure 1—figure supplement 1E, tested in single-pair assays with mated females as trainers and testers. Courtship indices (CIs) are shown as median of n males and dispersion of the data as interquartile range (IQR). P values determined by permutation test for the null hypothesis that learning equals 0 (H0: SI = 0) or for the null hypothesis that experimental and control males learn equally well (H0: SI = SIc). Table S2. dopR1 mutant court mated females more than wild-type males Courtship indices (CIs) of naïve males of the indicated genotypes according to Figure 1—figure supplement 1G during 6 hr training with mated female are shown as median of n males and dispersion of the data as interquartile range (IQR). P values determined by permutation test for the null hypothesis that CIs of both groups are equal (H0: CIwt = CIDopR1). Table S3. dopR1 mutant males do not learn Suppression indices (SIs) of naïve (train-) and experienced (train+) males of the indicated genotypes according to Figure 1—figure supplement 1H, tested in single-pair assays with mated females as trainers and testers. Courtship indices (CIs) are shown as median of n males and dispersion of the data as interquartile range (IQR). P values determined by permutation test for the null hypothesis that learning equals 0 (H0: SI = 0) or for the null hypothesis that experimental and control males learn equally well (H0: SI = SIc). Table S4. Day-time sleep deprivation between 7–9 hr impairs LTM Suppression indices (SIs) of naïve (train-) and experienced (train+) males of the indicated genotypes, sleep deprived as denoted in Figure 2A, tested in single-pair assays with mated females as trainers and testers. Courtship indices (CIs) are shown as median of n males and dispersion of the data as interquartile range (IQR). P values determined [file elife-42786-supp1.docx]

**Supplementary File 1. Tables S1-10**

**Table S1. LTM does not depend on the circadian time of training**

|  | **Genotype** | **Train**  **(0-6h)** | **Train**  **(6-12h)** | **n** | **CI (%)**  Median  *IQR* | **SI (%)** | ***P SI=0*** | ***P SI_n_=SI_2_*** |
| --- | --- | --- | --- | --- | --- | --- | --- | --- |
| 1 | *Wild-type* | - | - | 48 | 42.83  *16.38* |  |  |  |
| 2 | *Wild-type* | + | - | 46 | 29.14  *21.98* | 31.97 | 0.0001 |  |
| 3 | *Wild-type* | - | - | 53 | 42.36  *17.91* |  |  |  |
| 4 | *Wild-type* | - | + | 50 | 28.01  *16.48* | 33.89 | 0 | 0.86 |

Suppression indices (SIs) of naïve (train-) and experienced (train+) males of the indicated genotypes according to Figure 1-figure supplement E, tested in single-pair assays with mated females as trainers and testers. Courtship indices (CIs) are shown as median of *n* males and dispersion of the data as interquartile range (*IQR*). *P* values determined by permutation test for the null hypothesis that learning equals 0 (H_0_: SI = 0) or for the null hypothesis that experimental and control males learn equally well (H_0_: SI = SI_c_).

**Table S2. *dopR1* mutant court mated females more than wild-type males**

|  | **Genotype** | **Train**  **(0-6 h)** | **n** | **CI (%)**  Median  *IQR* | ***P CI wild-type = P CI dopR1*** |
| --- | --- | --- | --- | --- | --- |
| 1 | Wild-type | **+** | 54 | 21.6  *7.1* |  |
| 2 | *dopR1* | **+** | 54 | 29.3  *10.3* | 0.0002 |

Courtship indices (CIs) of naïve males of the indicated genotypes according to Figure 1-figure supplement G during 6-hour training with mated female are shown as median of *n* males and dispersion of the data as interquartile range (*IQR*). *P* values determined by permutation test for the null hypothesis that CIs of both groups are equal (H_0_: CI_wt_ = CI_DopR1_).

**Table S3. *dopR1* mutant males do not learn**

|  | **Genotype** | **Train**  **(0-6 h)** | **n** | **CI (%)**  Median  *IQR* | **SI (%)** | ***P SI=0*** | ***P SI_4_=SI_2_*** |
| --- | --- | --- | --- | --- | --- | --- | --- |
| 1 | *Wild-type* | - | 32 | 44.19  *22.14* |  |  |  |
| 2 | *Wild-type* | + | 34 | 21.77  *16.92* | 50.73 | 0 |  |
| 3 | *dopR1* | *-* | 26 | 38.41  *19.55* |  |  |  |
| 4 | *dopR1* | + | 29 | 34.04  *16.60* | 11.37 | 0.09 | 0.0002 |

Suppression indices (SIs) of naïve (train-) and experienced (train+) males of the indicated genotypes according to Figure 1-figure supplement H, tested in single-pair assays with mated females as trainers and testers. Courtship indices (CIs) are shown as median of *n* males and dispersion of the data as interquartile range (*IQR*). *P* values determined by permutation test for the null hypothesis that learning equals 0 (H_0_: SI = 0) or for the null hypothesis that experimental and control males learn equally well (H_0_: SI = SI_c_).

**Table S4. Day-time sleep deprivation between 7-9 hours impairs LTM**

|  | **Genotype** | **Train**  **(0-6 h)** | **Sleep deprivation** | **n** | **CI (%)**  Median  *IQR* | **SI (%)** | ***P SI=0*** | ***P SI_n_=SI_2_*** |
| --- | --- | --- | --- | --- | --- | --- | --- | --- |
| 1 | *Wild-type* | - | - | 35 | 54.58  *19.43* |  |  |  |
| 2 | *Wild-type* | + | - | 34 | 34.28  *15.79* | 37.19 | 0 |  |
| 3 | *Wild-type* | - | 7-9h | 31 | 45.54  *17.23* |  |  |  |
| 4 | *Wild-type* | + | 7-9h | 27 | 44.07  *16.02* | 3.22 | 0.44 | 0.007 |
| 5 | *Wild-type* | - | 8-10h | 35 | 34.87  *16.59* |  |  |  |
| 6 | *Wild-type* | + | 8-10h | 34 | 32.29  *17.26* | 7.39 | 0.36 | 0.03 |
| 7 | *Wild-type* | - | 9-11h | 23 | 45.63  *26.33* |  |  |  |
| 8 | *Wild-type* | + | 9-11h | 26 | 33.91  *12.85* | 38.94 | 0.002 | 0.87 |
| 9 | *Wild-type* | - | 10-12h | 34 | 29.65  *14.51* |  |  |  |
| 10 | *Wild-type* | + | 10-12h | 36 | 19.70  *19.82* | 33.56 | 0.001 | 0.84 |

Suppression indices (SIs) of naïve (train-) and experienced (train+) males of the indicated genotypes, sleep deprived as denoted in Figure 2A, tested in single-pair assays with mated females as trainers and testers. Courtship indices (CIs) are shown as median of *n* males and dispersion of the data as interquartile range (*IQR*). *P* values determined by permutation test for the null hypothesis that learning equals 0 (H_0_: SI = 0) or for the null hypothesis that experimental and control males learn equally well (H_0_: SI = SI_c_).

**Table S5. Night-time sleep deprivation does not impair LTM**

|  | **Genotype** | **Train**  **(0-6 h)** | **Sleep deprivation** | **n** | **CI (%)**  Median  *IQR* | **SI (%)** | ***P SI=0*** | ***P SI_n_=SI_2_*** |
| --- | --- | --- | --- | --- | --- | --- | --- | --- |
| 1 | *Wild-type* | - | - | 36 | 43.80  *12.88* |  |  |  |
| 2 | *Wild-type* | + | - | 36 | 28.65  *24.26* | 34.59 | 0 |  |
| 3 | *Wild-type* | - | 12-14h | 36 | 43.42  *10.88* |  |  |  |
| 4 | *Wild-type* | + | 12-14h | 36 | 33.91  *17.00* | 21.90 | 0 | 0.18 |
| 5 | *Wild-type* | - | 14-16h | 36 | 38.08  *14.69* |  |  |  |
| 6 | *Wild-type* | + | 14-16h | 33 | 33.16  *11.51* | 12.94 | 0.017 | 0.034 |
| 7 | *Wild-type* | - | 16-18h | 35 | 36.68  *11.68* |  |  |  |
| 8 | *Wild-type* | + | 16-18h | 33 | 30.74  *16.80* | 16.18 | 0.014 | 0.097 |
| 9 | *Wild-type* | - | 18-20h | 34 | 34.81  *12.75* |  |  |  |
| 10 | *Wild-type* | + | 18-20h | 36 | 25.68  *10.58* | 26.24 | 0 | 0.42 |
| 11 | *Wild-type* | - | 20-22h | 36 | 38.10  *20.13* |  |  |  |
| 12 | *Wild-type* | + | 20-22h | 33 | 28.29  *20.94* | 25.76 | 0.01 | 0.46 |
| 13 | *Wild-type* | - | 22-24h | 33 | 42.50  *18.94* |  |  |  |
| 14 | *Wild-type* | + | 22-24h | 34 | 33.42  *16.67* | 21.35 | 0.018 | 0.21 |

Suppression indices (SIs) of naïve (train-) and experienced (train+) males of the indicated genotypes, sleep deprived as denoted in Figure 2-figure supplement A, tested in single-pair assays with mated females as trainers and testers. Courtship indices (CIs) are shown as median of *n* males and dispersion of the data as interquartile range (*IQR*). *P* values determined by permutation test for the null hypothesis that learning equals 0 (H_0_: SI = 0) or for the null hypothesis that experimental and control males learn equally well (H_0_: SI = SI_c_).

**Table S6. Silencing of DAN-aSP13 between 7-9 hours impairs LTM**

|  | **Genotype** | **Train**  **(0-6 h)** | **Silencing** | **n** | **CI (%)**  Median  *IQR* | **SI (%)** | ***P SI=0*** | ***P SI_n_=SI_2_*** |
| --- | --- | --- | --- | --- | --- | --- | --- | --- |
| 1 | *VT005526-LexA>*  *LexAop-shi^ts^* | - | - | 53 | 59.11  *27.37* |  |  |  |
| 2 | *VT005526-LexA>*  *LexAop-shi^ts^* | + | - | 53 | 37.01  *21.63* | 37.39 | 0 |  |
| 3 | *VT005526-LexA>*  *LexAop-shi^ts^* | - | 7-9h | 36 | 57.35  *15.63* |  |  |  |
| 4 | *VT005526-LexA>*  *LexAop-shi^ts^* | + | 7-9h | 35 | 53.63  *16.85* | 6.49 | 0.23 | 0.002 |
| 5 | *VT005526-LexA>*  *LexAop-shi^ts^* | - | 8-10h | 53 | 50.04  *12.43* |  |  |  |
| 6 | *VT005526-LexA>*  *LexAop-shi^ts^* | + | 8-10h | 54 | 49.26  *24.56* | 1.57 | 0.46 | 0 |
| 7 | *VT005526-LexA>*  *LexAop-shi^ts^* | - | 9-11h | 34 | 43.36  *20.29* |  |  |  |
| 8 | *VT005526-LexA>*  *LexAop-shi^ts^* | + | 9-11h | 32 | 31.12  *17.18* | 28.23 | 0.003 | 0.47 |
| 9 | *VT005526-LexA>*  *LexAop-shi^ts^* | - | 10-12h | 53 | 44.37  *24.25* |  |  |  |
| 10 | *VT005526-LexA>*  *LexAop-shi^ts^* | + | 10-12h | 54 | 36.25  *21.54* | 18.30 | 0.03 | 0.04 |

Suppression indices (SIs) of naïve (train-) and experienced (train+) males of the indicated genotypes with DAN-aSP13 silenced as denoted in Figure 2B, tested in single-pair assays with mated females as trainers and testers. Courtship indices (CIs) are shown as median of *n* males and dispersion of the data as interquartile range (*IQR*). *P* values determined by permutation test for the null hypothesis that learning equals 0 (H_0_: SI = 0) or for the null hypothesis that experimental and control males learn equally well (H_0_: SI = SI_c_).

**Table S7. Sleep induction between 5-7 hours consolidates STM to LTM**

|  | **Genotype** | **Train**  **(0-1 h)** | **Activation** | **n** | **CI (%)**  Median  *IQR* | **SI (%)** | ***P SI=0*** | ***P SI_n_=SI_2_*** |
| --- | --- | --- | --- | --- | --- | --- | --- | --- |
| 1 | *104y-GAL4>*  *UAS-CsChrimson* | - | - | 34 | 24.41  *17.67* |  |  |  |
| 2 | *104y-GAL4>*  *UAS-CsChrimson* | + | - | 33 | 26.79  *15.61* | -9.77 | 0.73 |  |
| 3 | *104y-GAL4>*  *UAS-CsChrimson* | - | 1-3h | 35 | 31.57  *16.22* |  |  |  |
| 4 | *104y-GAL4>*  *UAS-CsChrimson* | + | 1-3h | 34 | 31.22  *14.55* | 1.11 | 0.55 | 0.52 |
| 5 | *104y-GAL4>*  *UAS-CsChrimson* | - | 3-5h | 33 | 32.55  *19.94* |  |  |  |
| 6 | *104y-GAL4>*  *UAS-CsChrimson* | + | 3-5h | 35 | 30.90  *21.18* | 5.06 | 0.39 | 0.49 |
| 7 | *104y-GAL4>*  *UAS-CsChrimson* | - | 5-7h | 47 | 31.96  *16.07* |  |  |  |
| 8 | *104y-GAL4>*  *UAS-CsChrimson* | + | 5-7h | 48 | 22.76  *12.13* | 28.77 | 0.00047 | 0.0077 |
| 9 | *104y-GAL4>*  *UAS-CsChrimson* | - | 7-9h | 36 | 24.71  *18.62* |  |  |  |
| 10 | *104y-GAL4>*  *UAS-CsChrimson* | + | 7-9h | 33 | 25.02  *21.02* | -1.27 | 0.50 | 0.63 |
| 11 | *104y-GAL4>*  *UAS-CsChrimson* | - | 9-11h | 31 | 30.83  *22.64* |  |  |  |
| 12 | *104y-GAL4>*  *UAS-CsChrimson* | + | 9-11h | 32 | 27.14  *21.76* | 11.65 | 0.26 | 0.35 |
| 13 | *104y-GAL4>*  *UAS-CsChrimson;*  *VT005526-LexA>*  *LexAop-shi^ts^* | - | 5-7h | 32 | 45.78  *17.09* |  |  |  |
| 14 | *104y-Gal4>*  *UAS-CsChrimson;*  *VT005526-LexA>*  *LexAop-shi^ts^* | + | 5-7h | 31 | 40.12  *23.61* | 12.36 | 0.14 | 0.80 |

Suppression indices (SIs) of naïve (train-) and experienced (train+) males of the indicated genotypes and sleep induced as denoted in Figure 2C, tested in single-pair assays with mated females as trainers and testers. Courtship indices (CIs) are shown as median of *n* males and dispersion of the data as interquartile range (*IQR*). *P* values determined by permutation test for the null hypothesis that learning equals 0 (H_0_: SI = 0) or for the null hypothesis that experimental and control males learn equally well (H_0_: SI = SI_c_).

**Table S8. DAN-aSP13 activation between 5-7 hours consolidates LTM**

|  | **Genotype** | **Train**  **(0-1 h)** | **Activation** | **n** | **CI (%)**  Median  *IQR* | **SI (%)** | ***P SI=0*** | ***P SI_n_=SI_2_*** |
| --- | --- | --- | --- | --- | --- | --- | --- | --- |
| 1 | *VT005526-LexA>*  *LexAop-CsChrimson* | - | - | 53 | 36.20  *19.22* |  |  |  |
| 2 | *VT005526-LexA>*  *LexAop-CsChrimson* | + | - | 48 | 37.14  *17.04* | -2.60 | 0.66 |  |
| 3 | *VT005526-LexA> LexAop-CsChrimson* | - | 1-3h | 34 | 30.74  *25.39* |  |  |  |
| 4 | *VT005526-LexA> LexAop-CsChrimson* | + | 1-3h | 33 | 26.61  *20.53* | 13.44 | 0.17 | 0.28 |
| 5 | *VT005526-LexA> LexAop-CsChrimson* | - | 3-5h | 34 | 39.48  *18.42* |  |  |  |
| 6 | *VT005526-LexA> LexAop-CsChrimson* | + | 3-5h | 26 | 41.00  *24.92* | -3.85 | 0.66 | 0.94 |
| 7 | *VT005526LexA> LexAop-CsChrimson* | - | 5-7h | 49 | 36.39  *22.80* |  |  |  |
| 8 | *VT005526-LexA> LexAop-CsChrimson* | + | 5-7h | 46 | 24.39  *20.55* | 32.96 | 0.002 | 0.009 |
| 9 | *VT005526-LexA> LexAop-CsChrimson* | - | 7-9h | 53 | 33.89  *12.74* |  |  |  |
| 10 | *VT005526-LexA> LexAop-CsChrimson* | + | 7-9h | 43 | 35.86  *8.56* | -5.82 | 0.85 | 0.79 |
| 11 | *VT005526-LexA> LexAop-CsChrimson* | - | 9-11h | 50 | 32.10  *14.20* |  |  |  |
| 12 | *VT005526-LexA> LexAop-CsChrimson* | + | 9-11h | 47 | 29.46  *14.43* | 8.22 | 0.08 | 0.50 |

Suppression indices (SIs) of naïve (train-) and experienced (train+) males of the indicated genotypes with DAN-aSP13 activated as denoted in Figure 2D, tested in single-pair assays with mated females as trainers and testers. Courtship indices (CIs) are shown as median of *n* males and dispersion of the data as interquartile range (*IQR*). *P* values determined by permutation test for the null hypothesis that learning equals 0 (H_0_: SI = 0) or for the null hypothesis that experimental and control males learn equally well (H_0_: SI = SI_c_).

**Table S9. Activation of vFB neurons between 5-7 hours consolidates LTM**

|  | **Genotype** | **Train**  **(0-1 h)** | **Activation** | **n** | **CI (%)**  Median  *IQR* | **SI (%)** | ***P SI=0*** | ***P SI_act_=SI_c_*** |
| --- | --- | --- | --- | --- | --- | --- | --- | --- |
| 1 | *104y-GAL4>*  *UAS-CsChrimson* | - | - | 50 | 46.79  *18.91* |  |  |  |
| 2 | *104y-GAL4>*  *UAS-CsChrimson* | + | - | 48 | 43.41  *16.46* | 7.21 | 0.11 |  |
| 3 | *104y-GAL4>*  *UAS-CsChrimson* | - | 5-7h | 51 | 49.42  *18.44* |  |  |  |
| 4 | *104y-GAL4>*  *UAS-CsChrimson* | + | 5-7h | 49 | 35.60  *15.89* | 27.96 | 0 | 0.02 |
| 5 | *R23E10-GAL4>*  *UAS-CsChrimson* | - | ­­­­­­­­­- | 33 | 58.36  *11.26* |  |  |  |
| 6 | *R23E10-GAL4>*  *UAS-CsChrimson* | + | - | 31 | 52.68  *11.57* | 9.72 | 0.01 |  |
| 7 | *R23E10-GAL4>*  *UAS-CsChrimson* | - | 5-7h | 33 | 52.52  *20.43* |  |  |  |
| 8 | *R23E10-GAL4>*  *UAS-CsChrimson* | + | 5-7h | 32 | 50.72  *13.77* | 3.41 | 0.40 | 0.52 |
| 9 | *VT036875-GAL4>*  *UAS-CsChrimson* | - | - | 36 | 27.84  *12.09* |  |  |  |
| 10 | *VT036875-GAL4>*  *UAS-CsChrimson* | + | - | 30 | 32,11  *17.99* | -15.33 | 0.95 |  |
| 11 | *VT036875-GAL4>*  *UAS-CsChrimson* | - | 5-7h | 36 | 32.54  *17.53* |  |  |  |
| 12 | *VT036875-GAL4>*  *UAS-CsChrimson* | + | 5-7h | 28 | 23.80  *22.06* | 26.85 | 0.04 | 0.031 |
| 13 | *VT036875-GAL4>*  *UAS-CsChrimson, R58E02-GAL80* | - | - | 48 | 34.75  *20.01* |  |  |  |
| 14 | *VT036875-GAL4>*  *UAS-CsChrimson, R58E02-GAL80* | + | - | 52 | 36,78  *25.53* | -5.85 | 0.67 |  |
| 15 | *VT036875-GAL4>*  *UAS-CsChrimson, R58E02-GAL80* | - | 5-7h | 47 | 34.40  *18.75* |  |  |  |
| 16 | *VT036875-GAL4>*  *UAS-CsChrimson, R58E02-GAL80* | + | 5-7h | 46 | 26.78  *18.50* | 22.17 | 0.0026 | 0.031 |
| 17 | *SS57264-GAL4>*  *UAS-CsChrimson* | - | - | 53 | 39.33  *18.15* |  |  |  |
| 18 | *SS57264-GAL4>*  *UAS-CsChrimson* | + | - | 48 | 41.43  *26.89* | -5.33 | 0.77 |  |
| 19 | *SS57264-GAL4>*  *UAS-CsChrimson* | - | 5-7h | 54 | 38.41  *17.35* |  |  |  |
| 20 | *SS57264-GAL4>*  *UAS-CsChrimson* | + | 5-7h | 47 | 28.77  *22.06* | 25.10 | 0.0008 | 0.038 |
| 21 | *pBDP-GAL4>*  *UAS-CsChrimson* | - | 5-7h | 33 | 36.32  *14.02* |  |  |  |
| 22 | *pBDP-GAL4>*  *UAS-CsChrimson* | + | 5-7h | 32 | 40.15  *13.86* | -10.53 | 0.96 |  |

Suppression indices (SIs) of naïve (train-) and experienced (train+) males of the indicated genotypes with vFB activated as denoted in Figure 5A, tested in single-pair assays with mated females as trainers and testers. Courtship indices (CIs) are shown as median of *n* males and dispersion of the data as interquartile range (*IQR*). *P* values determined by permutation test for the null hypothesis that learning equals 0 (H_0_: SI = 0) or for the null hypothesis that experimental and control males learn equally well (H_0_: SI = SI_c_).

**Table S10. Silencing of vFB neurons between 7-10 hours impairs LTM**

|  | **Genotype** | **Train**  **(0-6 h)** | **Silencing** | **n** | **CI (%)**  Median  *IQR* | **SI (%)** | ***P SI=0*** | ***P SI_sil_=SI_c_*** | ***P SI=SI_empty_*** |
| --- | --- | --- | --- | --- | --- | --- | --- | --- | --- |
| 1 | *104y-GAL4>*  *UAS-shi^ts^* | - | - | 61 | 48.72  *27.83* |  |  |  |  |
| 2 | *104y-GAL4>*  *UAS-shi^ts^* | + | - | 63 | 36.81  *32.47* | 24.44 | 0.006 |  | 0.61 |
| 3 | *104y-GAL4>*  *UAS-shi^ts^* | - | 7-10h | 54 | 30.76  *26.96* |  |  |  |  |
| 4 | *104y-GAL4>*  *UAS-shi^ts^* | + | 7-10h | 55 | 34.64  *30.94* | -12.64 | 0.70 | 0.043 | 0.049 |
| 5 | *R23E10-GAL4>*  *UAS-shi^ts^* | - | - | 48 | 62.29  *12.23* |  |  |  |  |
| 6 | *R23E10-GAL4>*  *UAS-shi^ts^* | + | - | 49 | 52.90  *12.84* | 15.06 | 0.0003 |  | 0.15 |
| 7 | *R23E10-GAL4>*  *UAS-shi^ts^* | - | 7-10h | 51 | 60.19  *19.75* |  |  |  |  |
| 8 | *R23E10-GAL4>*  *UAS-shi^ts^* | + | 7-10h | 53 | 49.70  *14.96* | 17.43 | 0 | 0.65 | 0.21 |
| 9 | *VT036875-GAL4>*  *UAS-shi^ts^* | - | - | 31 | 48.40  *12.46* |  |  |  |  |
| 10 | *VT036875-GAL4>*  *UAS-shi^ts^* | + | - | 32 | 41.48  *14.33* | 14.42 | 0.007 |  | 0.026 |
| 11 | *VT036875-GAL4>*  *UAS-shi^ts^* | - | 7-10h | 31 | 44.02  *12.53* |  |  |  |  |
| 12 | *VT036875-GAL4>*  *UAS-shi^ts^* | + | 7-10h | 28 | 45.00  *18.10* | -8.37 | 0.85 | 0.03 | 0.0075 |
| 13 | *SS57264-GAL4>*  *UAS-shi^ts^* | - | - | 36 | 51.76  *11.68* |  |  |  |  |
| 14 | *SS57264-GAL4>*  *UAS-shi^ts^* | + | - | 36 | 43.85  *20.48* | 15.28 | 0.011 |  | 0.080 |
| 15 | *SS57264-GAL4>*  *UAS-shi^ts^* | - | 7-10h | 35 | 47.28  *12.24* |  |  |  |  |
| 16 | *SS57264-GAL4>*  *UAS-shi^ts^* | + | 7-10h | 36 | 47.15  *18.33* | 0.28 | 0.50 | 0.03 | 0.0016 |
| 17 | *pBDP-GAL4>*  *UAS-CsChrimson* | - | 7-10h | 28 | 44.91  *12.43* |  |  |  |  |
| 18 | *pBDP-GAL4>*  *UAS-CsChrimson* | + | 7-10h | 25 | 30.66  *12.89* | 31.7 | 0.0004 |  |  |

Suppression indices (SIs) of naïve (train-) and experienced (train+) males of the indicated genotypes with vFB silenced as denoted in Figure 5B, tested in single-pair assays with mated females as trainers and testers. Courtship indices (CIs) are shown as median of *n* males and dispersion of the data as interquartile range (*IQR*). *P* values determined by permutation test for the null hypothesis that learning equals 0 (H_0_: SI = 0) or for the null hypothesis that experimental and control males learn equally well (H_0_: SI = SI_c_).
