# Supplementary material for: Neuronal reactivation during post-learning sleep consolidates long-term memory in Drosophila
Source: eLife. 2019 Feb 25;8:e42786. doi: 10.7554/eLife.42786 (PMC6428568; doi:10.7554/eLife.42786)
Supplement: Supplementary file 2. — Specific fly genotypes used in all main and supplementary figures. [file elife-42786-supp2.docx]

**Supplementary File 2. Fly genotypes**

**Figure 1A**

*+/Y; UAS-FLP.PEST/MB315B-ZpGAL4dbd; lola>>luc/MB315B-GAL4p65adz*

*+/Y; UAS-FLP.PEST/+; lola>>luc/+*

Trainer females: Canton-S, mated to Canton-S males

**Figure 1B**

*+/Y; +; +* (Canton-S)

Trainer females: Canton-S, mated to Canton-S males

**Figure 1C**

*+/Y; +; dopR1attp*

Trainer females: Canton-S, mated to Canton-S males

**Figure 1 - Figure supplement 1A**

+/Y; *MB315B-ZpGAL4dbd; MB315B-GAL4p65adz/ UAS-myr::smGFP*

**Figure 1 - Figure supplement 1B**

*+/Y; UAS-FLP.PEST/MB315B-ZpGAL4dbd; lola>>luc/MB315B-GAL4p65adz*

*+/Y; UAS-FLP.PEST/+; lola>>luc/+*

Trainer females: Canton-S virgin females

**Figure 1 - Figure supplement 1C**

*+/Y; UAS-FLP.PEST/MB315B-ZpGAL4dbd; lola>>luc/MB315B-GAL4p65adz*

*+/Y; UAS-FLP.PEST/+; lola>>luc/+*

Trainer females: Canton-S, mated to Canton-S males

**Figure 1 - Figure supplement 1D**

*+/Y; +; +* (Canton-S)

Trainer females: Canton-S, mated to Canton-S males

**Figure 1 - Figure supplement 1E**

*+/Y; +; +* (Canton-S)

Trainer and tester females: Canton-S, mated to Canton-S males

**Figure 1 - Figure supplement 1F**

*+/Y; +; +* (Canton-S)

Trainer females: Canton-S

**Figure 1 - Figure supplement 1G**

*+/Y; +; +* (Canton-S)

*+/Y; +; dopR1attp*

Trainer and tester females: Canton-S, mated to Canton-S males

**Figure 1 - Figure supplement 1H**

*+/Y; +; +* (Canton-S)

*+/Y; +; dopR1attp*

Trainer and tester females: Canton-S, mated to Canton-S males

**Figure 2A**

*+/Y; +; +* (Canton-S)

Trainer and tester females: Canton-S, mated to Canton-S males

**Figure 2B**

*+/Y; VT005526-LexAGAD/+; LexAop-shi^ts^/+*

Trainer and tester females: Canton-S, mated to Canton-S males

**Figure 2C**

*+/Y; 104y-GAL4/+; UAS-CsChrimson/+*

*+/Y; 104y-GAL4/VT005526-LexAGAD; UAS-CsChrimson/LexAop-shi^ts^*

Trainer females: Canton-S, mated to Canton-S males

**Figure 2D**

*+/Y; VT005526-LexAGAD/+; LexAop2-CsChrimson/+*

Trainer and tester females: Canton-S, mated to Canton-S males

**Figure 2 - Figure supplement A**

*+/Y; +; +* (Canton-S)

Trainer and tester females: Canton-S, mated to Canton-S males

**Figure 2 - Figure supplement 2B**

+/Y; *VT005526-LexA/+;LexAop--myr::smGFP/+*

**Figure 2 - Figure supplement 2C**

*+/Y; 104y-GAL4/+; UAS-dTrpA1/+*

*+/Y; pBDP-GAL4/+; UAS-dTrpA1/+*

**Figure2 - figure supplement 2D**

*+/Y; VT005526-LexAGAD/+; LexAop2-CsChrimson/+*

Trainer and tester females: Canton-S, mated to Canton-S males

**Figure 3A**

*UAS-Chrimson88, UAS-GCaMP6s/Y; 104y-GAL4/+; +/+*

**Figure 3B, 3C and 3D**

*UAS-Chrimson88, LexAop-GCaMP6s/Y; R58E02-LexAp65/104y-GAL4; +/+*

**Figure 3 - Figure supplement 3A**

*LexAop-GCaMP6s/Y; R58E02-LexA/+; +/+*

**Figure 3 - Figure supplement 3B**

*UAS-Chrimson88, LexAop-GCaMP6s/Y; R58E02-LexAp65/104y-GAL4; +/+*

**Figure 4A**

*+/Y; +/+; UAS-dTrpA1/R23E10-GAL4*

*UAS-Chrimson88, LexAop-GCaMP6s/Y; R58E02-LexAp65/R23E10-GAL4; +/+*

**Figure 4B**

*+/Y; +/+; UAS-dTrpA1/VT036875-GAL4*

*UAS-Chrimson88, LexAop-GCaMP6s/Y; R58E02-LexAp65/+; VT036875-GAL4/+*

**Figure 4C**

*+/Y; +/+; UAS-dTrpA1/+; VT036875-GAL4/R58E02-GAL80*

*UAS-Chrimson88*, *LexAop-GCaMP6s/Y; R58E02-LexAp65/+; VT036875-GAL4/R58E02-GAL80*

**Figure 4D**

*+/Y; +/+; UAS-dTrpA1/SS57264-GAL4p65adz; SS57264-ZpGAL4dbd/+*

*UAS-Chrimson88*, *LexAop-GCaMP6s/Y; R58E02-LexAp65/SS57264-GAL4p65adz; SS57264-ZpGAL4dbd/+*

**Figure 4 - figure supplement B**

*+/Y; + /+; R23E10-GAL4/UAS-myr::smGFP*

**Figure 4 - figure supplement C**

*+/Y; +/+; VT036875-GAL4/UAS-myr::smGFP*

**Figure 4 - figure supplement D**

*UAS-Chrimson88-tdTomato/Y; +/+; VT036875-GAL4/R58E02-GAL80*

**Figure 4 - figure supplement E**

*UAS-Chrimson88-tdTomato/Y; +/SS57264-GAL4p65adz; SS57264-ZpGAL4dbd/+*

**Figure 4 - Figure supplement F**

*+/Y; + /+; R23E10-GAL4/UAS-CsChrimson*

*+/Y; +/+; VT036875-GAL4/UAS-CsChrimson*

*UAS-CsChrimson/Y; +/+; VT036875-GAL4/R58E02-GAL80*

*UAS-CsChrimson/Y; +/SS57264-GAL4p65adz; SS57264-ZpGAL4dbd/+*

*+/Y; 104y-GAL4/+; UAS-CsChrimson/+*

*+/Y; 104y-GAL4/+; UAS-CsChrimson/+*

*UAS-CsChrimson/Y; +/+; pBDP-GAL4/+*

**Figure 4 - Figure supplement G**

*+/Y; UAS-dTrpA1/+; pBDP-GAL4/+*

**Figure 5A**

*UAS-CsChrimson/Y; +/104y-GAL4; +/+*

*UAS-CsChrimson/Y; +/+; R23E10-GAL4/+*

*UAS-CsChrimson/Y; +/+; VT036875-Gal4/+*

*UAS-CsChrimson/Y; +/+; VT036875-GAL4/R58E02-GAL80*

*UAS-CsChrimson/Y; +/SS57264-GAL4p65adz; SS57264-ZpGAL4dbd/+*

*UAS-CsChrimson/Y; +/+; pBDP-GAL4/+*

Trainer and tester females: Canton-S, mated to Canton-S males

**Figure 5B**

*+/Y; 104y-GAL4/UAS-shi^ts^; +/+*

*+/Y; UAS-shi^ts^/+; R23E10-GAL4/+*

*+/Y; UAS-shi^ts^/+; VT036875-GAL4/+*

*+/Y; UAS-shi^ts^/SS57264-GAL4p65adz; SS57264-ZpGAL4dbd /+*

*+/Y; UAS-shi^ts^/+; pBDP-GAL4/+*

Trainer and tester females: Canton-S, mated to Canton-S males
